# Skipping breakfast among 8-9 year old children is associated with teacher-reported but not objectively measured academic performance two years later

**DOI:** 10.1186/s40795-017-0205-8

**Published:** 2017-12-06

**Authors:** Kylie J. Smith, Leigh Blizzard, Sarah A. McNaughton, Seana L. Gall, Monique C. Breslin, Melissa Wake, Alison J. Venn

**Affiliations:** 1 Menzies Institute for Medical Research, University of Tasmania, Private Bag 23, Hobart, TAS 7000 Australia; 2 Institute for Physical Activity and Nutrition, School of Exercise and Nutrition Sciences, Deakin University, Melbourne, Australia; 3 Murdoch Childrens Research Institute, Melbourne, Australia; 4 Department of Paediatrics, The University of Melbourne, Melbourne, Australia; 5 Department of Paediatrics and The Liggins Institute, The University of Auckland, Auckland, New Zealand

**Keywords:** Skipping breakfast, Academic performance, Behavior, Longitudinal, School, Breakfast

## Abstract

**Background:**

Skipping breakfast, habitually and when experimentally manipulated, has been linked in the short-term to poorer academic performance in children. Little is known about the longer-term effects. This study examined whether skipping breakfast at aged 8-9 years predicted poorer academic performance and classroom behavior 2 years later.

**Methods:**

The Longitudinal Study of Australian Children (LSAC) collected data during 2008 (aged 8-9 years) and 2010 (aged 10-11 years). Breakfast consumption was reported by a parent/caregiver on three occasions within 4 weeks during 2008: by face-to-face interview and two subsequent questionnaires. Children who skipped breakfast on at least one of the 3 days were classified as breakfast skippers. During 2010, the child’s teacher assessed their academic performance relative to other children in the same grade (below/far below average; average; above/far above average) and classroom behavior. Objective literacy and numeracy outcomes (reading, writing, spelling, grammar and numeracy, score range 0-1000) were obtained via linkage to Australian standardized national assessment program (NAPLAN) data in Year 5 (aged 10-11 years). Ordinal and linear regression were used, adjusted for sex, age and sociodemographic variables.

**Results:**

At baseline, 243 (10.7%) of the 2280 children skipped breakfast on at least 1 day. Two years later, breakfast skippers were more likely to have poorer teacher-reported reading (RR: 1.18; 95% CI: 1.08, 1.29), mathematics (RR: 1.11; 95% CI: 1.02, 1.20) and overall academic achievement (RR: 1.15; 95% CI: 1.05, 1.25) than non-skippers. In contrast, differences in objective NAPLAN scores were small (<3%), and only one of the five scales (numeracy) was significantly lower among skippers (mean difference − 13.0; 95% CI: -25.6, −0.8). Classroom behavior was similar between skippers and non-skippers.

**Conclusion:**

In this national sample of 8-9 year old Australian children, skipping breakfast occurred at low levels, and showed little association with measured academic performance 2 years later. This contrasted with teacher perceptions of lower academic performance among skippers than non-skippers, most likely reflecting confounding. This underscores the importance of using objective measures of academic performance to avoid inflated effect estimates and, potentially, unnecessary and costly population interventions to increase breakfast consumption.

**Electronic supplementary material:**

The online version of this article (10.1186/s40795-017-0205-8) contains supplementary material, which is available to authorized users.

## Background

Skipping breakfast has been linked to reductions in cognition and academic performance [1–3], resulting in strong arguments for public health interventions to increase breakfast consumption. However, well-designed randomized controlled trials (RCTs) [4–6] do not provide convincing evidence of benefit to support the substantial costs of school breakfast programs.

Experimental evidence indicates that, on any given day, skipping breakfast lowers cognitive and academic performance [3]. A systematic review, published in 2009, included 45 studies that examined the association between skipping breakfast and cognitive performance, including memory, attention and test grades. The review focused on school-aged children but the age of participants ranged from 3 years to a mean age of 21 years. The authors concluded that eating breakfast generally had a positive effect on cognitive performance [2]. The beneficial effects of eating breakfast were stronger when testing occurred later in the morning, when tests were more demanding and error rate was considered, and in malnourished populations [2]. In most studies, cognitive performance was assessed within 12 h of eating/skipping breakfast [2].

The long-term effects of skipping breakfast are less clear. A 2013 review examined the association between skipping breakfast and classroom behavior or academic performance (school grades or standardized achievement tests) among children and adolescents (aged 5 to 19 years) [1]. Five of the eight studies that examined habitual breakfast consumption reported a beneficial association with academic performance. Only one study used a longitudinal design [7], involving 21,400 5-year old USA children followed for 10 years. In contrast to the cross-sectional studies, frequency of breakfast consumption was not significantly associated with standardized test scores for reading, mathematics and science or teacher-reported behavior after adjusting for a wide range of potential confounders [7]. However, breakfast was defined as eating breakfast with the family, so children who ate breakfast alone or at school would be misclassified as breakfast skippers.

School breakfast programs are being implemented to increase breakfast consumption. Despite their popularity, there is little evidence to support their effectiveness. Three well-designed trials found programs that made school breakfast available to all children, did not improve academic achievement or classroom behavior among elementary school children (aged 4-14 years) [4–6]. However, none of the trials increased breakfast consumption among children who normally skipped breakfast; instead, children who usually ate breakfast at home ate it at school.

RCTs are the gold standard for determining causation but pose substantial challenges in this field. Most cross-sectional studies did not control for socioeconomic status (SES) [1], which is an important predictor of both academic performance [8] and breakfast consumption [9, 10]. Data examining the long-term effect of breakfast consumption on academic performance are sparse. Using data from a national study of Australian children, this study examines whether skipping breakfast at age 8-9 years predicts poorer academic performance and classroom behavior 2 years later. We hypothesize that breakfast skippers will have poorer school performance and classroom behavior than those who eat breakfast.

## Methods

Children were recruited for *Growing up in Australia*: the Longitudinal Study of Australian Children (LSAC) during 2004, aged 4-5 years (Wave 1, *N* = 4983). The sampling frame was extracted from the enrolment database of Medicare, the national health care scheme in Australia that enrolls 98% of Australian residents by 1 year of age [11]. The children were selected using a two-stage design [11]. In the first stage, postcodes were stratified by state/territory and urban/rural location to ensure geographical representation. Very remote postcodes were excluded. Postcodes were then randomly selected. In the second stage, a 10% sample of children born between March 1999 and February 2000 were randomly selected. For each postcode, children were listed according to date of birth, and a systematic random sample was taken from this list to ensure a representative range of birth dates.

**Fig. 1:**
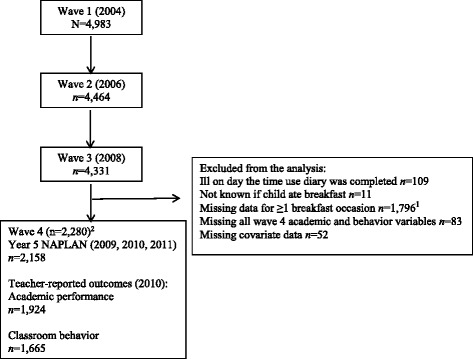
Participation at each wave of the Longitudinal Study of Australian Children and the final sample. ^1^1488 of these children (those with breakfast data from the interview and one diary) were included in a sensitivity analysis. ^2^Numbers for each outcome do not equal the total sample number due to missing data

The response rate at baseline was 54% (*N* = 4983). Data are collected biennially, with a core face-to-face home visit supplemented by a range of additional measures and data linkages. Data used in this analysis were collected via three mechanisms: home-based face-to-face interview and two subsequent written questionnaires completed by a parent/caregiver at wave 3 (2008); questionnaires completed by teachers at wave 3 (2008) and wave 4 (2010); and linked data from the Australian National Assessment Program - Literacy And Numeracy (NAPLAN), the national standardized school assessment program (Additional file 1: Figure S1). The children were 8-9 years old at wave 3 (participation rate 87% of wave 1) and 10-11 years at wave 4 (participation rate 84% of wave 1, Fig. 1).

The analysis of confidentialized LSAC data had the approval of the Australian Institute of Family Studies ethic committee. Consent to participate in the study was given by a parent at wave 1 and permission to link with the child’s NAPLAN results at wave 3 (or wave 4, if the child did not participate in wave 3).

### Breakfast (wave 3)

Breakfast consumption was assessed on three occasions, within 4 weeks, at wave 3 (baseline for this analysis). At the face-to-face interview, the parent/caregiver was asked “Did <study child> eat breakfast today?” After the interview, parents were asked to complete two time-use diaries on specified days the following week (one weekday and one weekend day), to which was appended a set of short dietary questions including whether the child had eaten breakfast that day. If the diary was not completed on the allocated date, the parent was asked to wait until the same day the following week. Parents reported whether the child was ill the day the diary was completed. The diaries were collected by the interviewer in person or returned by post. No data on breakfast consumption were collected at wave 4.

Children who skipped breakfast on at least one of the 3 days were classified as breakfast skippers and compared with those who ate breakfast on all three occasions (non-skippers). Further categorization was not possible because so few children skipped breakfast more than once (*n* = 17).

### Academic performance (wave 4 teacher report, year 5 academic testing)

A questionnaire was sent to the child’s teacher as soon as feasible after the wave 4 home interview [12], and on average, was completed 2 months after the interview. Questionnaires were completed for 3269 children (response rate 75.6%). The teacher was asked to compare the child’s reading, mathematics and overall progress to other children of the same level, on a 5-point scale. Response options were collapsed into three categories for the analysis: “far below/below average”, “average”, “above/far above average”.

NAPLAN assesses all Australian students in Years 3, 5, 7 and 9 (aged 8-9, 10-11, 12-13, and 14-15 years, respectively) across four domains: reading, writing, language conventions (spelling, grammar and punctuation), and numeracy, using national tests held on the same day in May across Australia each year. Scores are standardized to range from 0 to 1000 for each test, enabling comparisons within and across school year levels [13]. NAPLAN data for 4159 children were linked to the LSAC dataset (98.4% of those who consented to NAPLAN access, 83.5% of the total sample at wave 1). Of those who were not matched, 552 were not asked for consent as they did not participate in wave 3 or wave 4, 117 forms were completed incorrectly, 68 could not be linked and 48 refused permission [13]. Because the children did not all begin school in the same year, the Year 5 NAPLAN tests were completed in 2009, 2010 or 2011. For children who repeated a grade and sat the same NAPLAN test more than once, the latest score was used. NAPLAN data were available for 2158 children included in this analysis.

### Classroom behavior (wave 4)

The child’s teacher was asked to complete the Strengths and Difficulties Questionnaire (SDQ, Robert Goodman 1999, UK) in relation to the child’s classroom behavior. The SDQ includes 25 attributes, with the response options ‘not true’, ‘somewhat true’ and ‘certainly true’. The three scales recommended for low-risk or general population samples were used: internalizing problems (emotional + peer symptoms, 10 items, range 0-20), externalizing problems (conduct + hyperactivity symptoms, 10 items, range 0-20) and prosocial behavior (5 items, range 0-10) [14]. Lower scores for internalizing and externalizing problems and a higher score for prosocial behavior indicate better behavior. Children missing any of the subscale scores were excluded from the analysis.

### Covariates (wave 3)

Covariates considered for inclusion in the adjusted models included sociodemographic variables associated with skipping breakfast (described below) and the other outcome variables (teacher-reported performance, classroom behavior, and the standardized tests; for example the behavior variables were considered as covariates in the analysis examining the association between skipping breakfast and academic performance). The child’s age in months was recorded at the face-to-face interview and the NAPLAN tests. A continuous variable for SES was created from standardized scores for three variables: parents’ years of education; parents’ occupation as determined by the status of their main occupation; and combined annual income including pensions and allowances before tax (with natural log transformation) [15]. The primary caregiver reported their own health (excellent, very good, good, fair, poor); whether they currently smoked cigarettes (yes/no), whether the child had two parents living at home (yes/no) and the child’s ethnicity. Financial hardship was assessed using the Family Hardship Scale, which sums the number of positive responses to seven indicators of household hardship. To calculate the Family Hardship Scales, parent/caregivers were asked to report if they had experienced any of the following situations in the previous 12 months because they were short of money: inability to pay bills on time; unable to pay mortgage or rent on time; went without meals; were unable to heat or cool the home; pawned or sold something because needed cash; sought assistance from welfare or community organization; unable to send child to kindergarten/preschool/child care for as much time as they would like (potential score 0-7) [16]. Ethnicity was considered as a potential confounder in the analysis, but was not included in any of the final models as it did not change the coefficient of breakfast skipping by at least 10% (our criterion for including a potential confounder [17]) when included in the model. Year 3 NAPLAN test results were used in the propensity model.

### Statistical analysis

Log-link ordinal regression [18] was used to compare the probability of being in a lower level of teacher-reported scholastic performance for skippers and non-skippers. Results are reported for the continuation ratio probability model because for this model a test of the constraint underlying the ordinal assumption was satisfied in most cases. Differences between skippers and non-skippers in mean scores on the behavior subscales and NAPLAN results were estimated using linear regression. Model 1 included adjustment for age and sex. The teacher reported outcomes were adjusted for age at the time of the face-to-face interview, and the NAPLAN results were adjusted for age at the time the child sat the test. Model 2 had additional adjustment for SES, which was associated with both skipping breakfast and poorer academic performance. The other covariates included in model 3 were those that changed the coefficient of the covariate for skipping breakfast by at least 10% [17].

Inverse propensity weighting was used to take into account data missing from the baseline sample. The propensity model included baseline variables associated with missingness: SES; the primary caregiver’s sex, self-reported health status, education level and smoking status; household income; two-parent home; financial hardship; change in primary caregiver, child academic achievement (teacher-report and Year 3 NAPLAN) and child school attendance at wave 3. To ensure a full set of weights, missing observations in the variables required by each propensity model were imputed using multiple imputations by chained equations [19]. Ten imputations were performed.

A sensitivity analysis was conducted using only two measures of breakfast (the parent interview and one time-use diary) to allow children who were missing one diary to be included in the analysis. For children who had both diaries, one diary was randomly selected. We also examined cross-sectional associations between skipping breakfast and the wave 3 teacher-reported outcomes (collected the same way as in wave 4), but not the national standardized tests because most children sat the tests (May 2008) before the breakfast data were collected (March-December 2008, Additional file 1: Figure S1).

All analyses were conducted using Stata SE (version 12.1, 2011, StataCorp, College Station, TX). *P*-values ≤0.05 were considered statistically significant.

## Results

Of the 4331 eligible children, 2280 were included in the main analysis (Fig. 1). About one quarter of the children completed the Year 5 NAPLAN tests in 2009 (*n* = 500, 23.0% of the non-skippers and 24.5% of the skippers), the majority in 2010 (*n* = 1551, 71.9% non-skippers, 71.6% skippers), and 106 in 2011 (5.0% non-skippers, 3.9% skippers).

Compared with children who were included in the main analysis, those not included were more likely to have skipped breakfast on the day of the face-to-face interview (2.8% versus 6.7%, respectively, Additional file 1: Table S1) and to have greater SES disadvantage, poorer teacher reported academic performance and behavior, and lower NAPLAN scores (Additional file 1: Table S1).

The prevalence of skipping breakfast was similar between boys and girls: 113 (9.6%) boys and 113 (10.3%) girls skipped on 1 day, 10 (0.9%) boys and six (0.6%) girls skipped on 2 days, and one boy (0.1%) skipped all 3 days. Compared with non-skippers, a higher proportion of breakfast skippers were Indigenous, or from one-parent families (Table 1). However, there were few Indigenous children in the study sample (*n* = 31, 1.4%) so these findings may not be generalizable to the Indigenous population.

**Table 1:** Baseline characteristics of breakfast skippers and breakfast consumers, aged 8-9 years (*N* = 2280)

	Never skipped^a^ (*n* = 2037)^b^		≥1 skip^a^ (*n* = 243)^b^		*P*-value^c^
Characteristic	*n*	(%)	*n*	(%)	
Child’s sex					
Male	1057	(89.5)	124	(10.5)	
Female	980	(89.2)	119	(10.8)	0.800
Ethnicity					
Non-Indigenous	2012	(89.5)	236	(10.5)	
Indigenous	24	(77.4)	7	(22.6)	0.030
Sex of primary caregiver					
Male	79	(86.8)	12	(13.2)	
Female	1958	(89.5)	231	(10.6)	0.425
Socio-economic status (SES)^d^					
Most disadvantaged SES quartile	398	(87.5)	58	(12.5)	
Least disadvantaged SES quartile	418	(90.9)	44	(9.1)	0.098
Mother completed high school					
No	673	(87.7)	94	(12.3)	
Yes	1353	(90.3)	146	(9.7)	0.066
Father completed high school					
No	764	(88.7)	98	(11.3)	
Yes	1070	(91.1)	109	(8.9)	0.082
Two parent home					
No	183	(82.4)	39	(17.6)	
Yes	1854	(90.1)	204	(9.9)	<0.001

^a^Breakfast consumption was reported by a parent/caregiver on three separate days: by face-to-face interview and two subsequent time use diaries

^b^Numbers do not always equal the total sample number due to missing data

^c^ *P*-values calculated by chi-square analyses

^d^Socioeconomic status quartiles are based on the distribution of the Longitudinal Study of Australian Children data

Mean ± SD follow-up time was 2.03 ± 0.18 years. In the adjusted analysis, children who skipped breakfast at baseline were 18% (reading), 11% (mathematics) and 15% (overall achievement) more likely to be in a lower category for all three teacher-reported education outcomes at follow-up (Table 2). Breakfast status was not associated with classroom behavior (Table 3).

**Table 2:** Longitudinal associations between skipping breakfast aged 8-9 years and teacher-reported academic performance aged 10-11 years (*N* = 1924)

Outcome and category of skipping breakfast	Above average^a^	Average^a^	Below average^a^	Model 1^b^	Model 2^c^	Model 3^d^
	*n* (%)	*n* (%)	*n* (%)	RR (95% CI)	RR (95% CI)	RR (95% CI)
Reading progress						
Never skipped	760 (44.5)	694 (40.6)	254 (14.9)	1.00 (ref)	1.00 (ref)	1.00 (ref)
≥ 1 skips	77 (37.4)	90 (43.7)	39 (18.9)	1.15 (1.05, 1.26)	1.14 (1.05, 1.24)	1.18 (1.08, 1.29)
*P*-value				0.002	0.002	0.001
Mathematics progress						
Never skipped	705 (41.6)	748 (44.2)	241 (14.2)	1.00 (ref)	1.00 (ref)	1.00 (ref)
≥ 1 skips	79 (38.5)	88 (42.9)	38 (18.5)	1.11 (1.01, 1.22)	1.11 (1.03, 1.22)	1.11 (1.02, 1.20)
*P*-value				0.024	0.011	0.017
Overall achievement						
Never skipped	732 (43.0)	777 (45.7)	192 (11.3)	1.00 (ref)	1.00 (ref)	---^e^
≥ 1 skips	80 (38.6)	93 (44.9)	34 (16.4)	1.14 (1.04, 1.25)	1.15 (1.05, 1.25)	---^e^
*P*-value				0.007	0.002	

^a^Comparisons are to other children of the same grade level, calculated using log-link ordinal regression. Below average = below/far below average; Above average = above/far above average

^b^Model 1: adjusted for sex and age at time of the parent interview

^c^Model 2: adjusted for sex, age at time of the parent interview and SES (measured at Wave 3)

^d^Model 3: Model 2 plus the following additional covariates *reading progress* – teacher reported prosocial behavior at W4; *mathematics progress* – financial hardship

^e^There was no model 3 for overall achievement as none of the additional covariates changed the coefficient of the covariate for skipping breakfast by at least 10%

**Table 3:** Longitudinal associations between skipping breakfast aged 8-9 years and teacher-reported behavior aged 10-11 years (*N* = 1665)

Behavior subscale and category of skipping breakfast			Model 1^b^	Model 2^c^	Model 3^d^
	*n*	Mean ± SD^a^	Diff (95% CI)^e^	Diff (95% CI)^e^	Diff (95% CI)^e^
Internalizing problems					
Never skipped	1481	2.22 ± 2.92	0 (ref)	0 (ref)	0 (ref)
≥ 1 skips	177	2.40 ± 3.03	0.35 (−0.30, 1.00)	0.28 (−0.32, 0.88)	0.17 (−0.74, 1.07)
*P*-value			0.29	0.36	0.72
Externalizing problems					
Never skipped	1481	2.85 ± 3.52	0 (ref)	0 (ref)	0 (ref)
≥ 1 skips	177	3.08 ± 3.69	1.73 (−1.28, 4.74)	1.13 (−1.46, 3.71)	0.14 (−0.37, 0.64)
*P*-value			0.26	0.39	0.60
Prosocial behavior					
Never skipped	1486	7.97 ± 2.17	0 (ref)	0 (ref)	0 (ref)
≥ 1 skips	179	7.66 ± 2.21	−0.31 (−0.58, −0.05)	−0.26 (−0.51, −0.01)	−0.26 (−0.56, 0.03)
*P*-value			0.02	0.04	0.08

^a^Values are the unadjusted mean ± SD score for the three scales of the Strengths and Difficulties Questionnaire. Better behavior is indicated by lower scores for internalizing problems (range 0 – 18) and externalizing problems (range 0 – 20) and higher scores for prosocial behavior (range 0 – 10)

^b^Model 1: adjusted for sex and age at interview

^c^Model 2: adjusted for sex, age at interview and SES (measured at Wave 3)

^d^Model 3: Model 2 plus the following additional covariates *internalizing problems* – two-parent home, self-reported health of primary caregiver, smoking status of primary caregiver, financial hardship, reading progress; *externalizing problems* – self-reported health of primary caregiver, smoking status of primary caregiver, financial hardship, reading progress; *prosocial behavior* – two parent home, reading progress

^e^Differences between breakfast skippers and breakfast eaters were calculated using linear regression

The mean scores for all Year 5 NAPLAN domains (potential score range 0-1000) were consistently marginally lower among skippers than non-skippers, with differences in scores ranging from 6.2 for writing to 18.6 for numeracy in the unadjusted analysis (Table 4). The associations were attenuated after adjusting for SES and other covariates. In the final model, the differences in scores between skippers and non-skippers were less than 3% (range 0.8 for writing to 13.0 for numeracy).

**Table 4:** Longitudinal associations between skipping breakfast aged 8-9 years and national standardized test (Year-5 NAPLAN) results (*N* = 2158)

NAPLAN variable and category of skipping breakfast			Model 1^b^	Model 2^c^	Model 3^d^
	*n*	Mean ± SD^a^	Diff (95% CI)^e^	Diff (95% CI)^e^	Diff (95% CI)^e^
Reading					
Never skipped	1923	520 ± 79.4	0.00 (ref)	0.00 (ref)	0.00 (ref)
≥ 1 skips	227	510 ± 82.1	−15.3 (−27.2, −3.3)	−10.7 (−21.4, 0.02)	−9.2 (−19.7, 1.3)
*P*-value			0.01	0.05	0.09
Writing					
Never skipped	1904	502 ± 69.2	0.00 (ref)	0.00 (ref)	0.00 (ref)
≥ 1 skips	225	500 ± 64.1	−6.2 (−15.5, 3.1)	−2.1 (−10.8, 6.5)	−0.8 (−9.5, 7.9)
*P*-value			0.19	0.63	0.86
Spelling					
Never skipped	1922	502 ± 65.8	0.00 (ref)	0.00 (ref)	0.00 (ref)
≥ 1 skips	227	496 ± 69.1	−10.3 (−20.8, 0.2)	−7.4 (−17.2, 2.5)	−6.0 (−15.8, 3.8)
*P*-value			0.05	0.14	0.23
Grammar					
Never skipped	1922	528 ± 79.2	0.00 (ref)	0.00 (ref)	0.00 (ref)
≥ 1 skips	227	520 ± 93.8	−17.4 (−31.8, −3.0)	−12.8 (−25.7, 0.1)	−10.7 (−23.6, 2.1)
*P*-value			0.02	0.05	0.10
Numeracy					
Never skipped	1917	513 ± 70.5	0.00 (ref)	0.00 (ref)	0.00 (ref)
≥ 1 skips	227	502 ± 84.1	−18.6 (−32.7, −4.5)	−14.2 (−26.9, −1.6)	−13.0 (−25.6, −0.8)
*P*-value			0.01	0.03	0.04

*NAPLAN* National Assessment Program – Literacy And Numeracy, *SES* socioeconomic status

^a^Values are the unadjusted mean ± SD score for each domain of the NAPLAN assessments. Possible score range 0-1000, higher scores indicate better academic performance

^b^Model 1: adjusted for age and sex

^c^Model 2: adjusted for age, sex and SES (measured at Wave 3)

^d^Model 3: Model 2 plus the following additional covariates: *Reading* – smoking status of primary caregiver; *Writing* – smoking status of primary caregiver, self-reported health of primary caregiver; *Spelling* smoking status of primary caregiver, two-parent home status; *Grammar* – smoking status of primary caregiver, financial hardship; *Numeracy* – smoking status of primary caregiver

^e^Differences between breakfast skippers and breakfast eaters were calculated using linear regression

### Sensitivity analysis

Defining breakfast status from two reports of breakfast (*n* = 2768 children, 7.4% were breakfast skippers) did not substantially change the magnitude or significance of the results (data not shown). Teacher-reported academic performance (Additional file 1: Table S2) and behavior (Additional file 1: Table S3) were similar between skippers and non-skippers in the cross-sectional analysis.

## Discussion

In this sample of Australian children, those who skipped breakfast aged 8-9 years tended to have poorer teacher-reported academic performance than non-skippers 2 years later. However objective standardized tests showed the differences were marginal, generally not statistically significant, and attenuated after adjusting for SES. Although a weakly significant difference between breakfast skippers and non-skippers was observed in one of the five NAPLAN scales (Numeracy, *p* = 0.04), the difference between the two groups was less than 3% and unlikely to be of great importance. Skipping breakfast was not associated with classroom behavior.

A meaningful difference for NAPLAN has not been defined. In the adjusted analysis, we found breakfast skippers had a 13-point lower score in the Year 5 numeracy test than non-skippers. Every year the national NAPLAN results are reported for each test, stratified by a variety of sociodemographic variables [20]. To put our observed difference into context, the national NAPLAN report showed a similar difference for sex, with the mean Year 5 numeracy score being 11 points higher among boys than girls [20]. Compared to children living in metropolitan areas, those from provincial areas had a 16-point lower numeracy score, and those from remote and very remote areas had a 31-33 point lower score [20]. Larger differences were observed in the national data for parent education, compared to those who had a parent with a university degree, mean Year 5 numeracy scores were 31 points lower among those whose parent had a diploma and 46 points lower among those whose parents had only completed year 12 [20].

This work builds on previous cross-sectional studies by examining the effect of skipping breakfast on academic performance 2 years later. National standardized tests gave an objective measure of academic performance, in addition to the teacher-reported outcomes. Data on skipping breakfast were collected over three non-consecutive days, allowing us to better capture intermittent skippers than would be possible with a single day’s measure. To reduce the possibility that skipping breakfast was due to feeling unwell, children were excluded from the analysis if they were reported to be ill on any of the days breakfast consumption was assessed using the time use diary. The large national sample is also a strength of this study.

There are several limitations that need to be considered. The proportion of children who skipped breakfast was low, with a very small number of children skipping breakfast on more than one occasion. Our results may not be generalizable to populations where children regularly go without breakfast. It is likely that skipping breakfast will increase in the LSAC cohort in adolescence for a range of psychosocial reasons and, with repeated follow-ups, different patterns of association may emerge. Stronger associations with academic performance may be observed in children who regularly skip breakfast. However, consistent skipping was very rare and intermittent skipping better reflects what was occurring in this age group. Breakfast data were collected over 3 days. While this allowed us to identify intermittent skippers better than would have been possible with data collected on just 1 day, this may still not fully reflect children’s usual breakfast habits. The children who usually skip breakfast one out of 3 days may go to school without breakfast one or 2 days each week but other children classified as skippers may go without breakfast less frequently. We were unable to examine change in breakfast habits in this analysis as breakfast consumption was not assessed at follow-up, and it is possible that children’s breakfast habits may have changed during the 2-year follow-up. Future research should examine whether becoming a breakfast skipper is associated with poorer performance on standardized tests or if becoming a breakfast eater is associated with improved standardized test results. Breakfast consumption was reported by parents and there is the possibility of misreporting due to social desirability bias. However, LSAC - a convenient data source to examine these hypotheses - did not have an overt focus on diet, reducing the likelihood of participants reporting socially desirable answers. It is recommended that parents report dietary intake for children up to 10 years of age to obtain reliable information [21]. We considered a range of potential confounders but there remains the possibility of residual confounding.

Our findings support those of Miller et al., who reported no significant differences between skippers and non-skippers in academic performance, assessed using standardized tests, or classroom behavior over 10 years among children from the USA [7]. In that study some children who ate breakfast may have been misclassified as breakfast skippers as breakfast was defined as eating breakfast with the family. To our knowledge, that is the only other study to examine the longer-term effect of skipping breakfast and it is reassuring to replicate the results in another country and a different education system.

There are several potential explanations as to why skipping breakfast was associated with poorer teacher-reported academic performance but not with the national standardized tests. Skipping breakfast was weakly associated with reading and numeracy in the national standardized tests but the associations were attenuated after adjusting for SES and other confounders. Residual confounding is a possible reason for the association between skipping breakfast and teacher-reported assessment, which was subjective and may have been influenced by other factors associated with skipping breakfast such as perceptions of the home environment and family support. This highlights the importance of using objective measures of academic performance. It is also possible that some children may perform differently in formal test situations than in other teacher-assessed classroom tests and assignments. The children were not all enrolled in the same year of school, therefore, the NAPLAN assessments were completed over 3 years. Reassuringly, the proportions of skippers and non-skippers who completed the NAPLAN tests during each year were very similar, so should not have affected the association between skipping breakfast and academic performance.

In Australia, the Government does not fund school food assistance programs, such as free or subsidized lunches. Some schools do have breakfast programs, which are often funded by non-Government organizations, such as the Red Cross and Foodbank Australia [22]. Program delivery such as frequency, foods provided and eligibility varies by school. It is not known whether children in this study participated in a school breakfast program. The breakfast question asked whether breakfast was consumed that day and should include breakfast consumed at home, school or other locations.

The prevalence of skipping breakfast was lower than in other international studies using national data [9, 10, 23] with only 10% of children skipping breakfast at least once. This could be explained by the young age of our cohort, as skipping breakfast has been shown to increase with age [9, 24] and previous studies included older children (aged 9-14 years old). It is difficult to compare studies due to the different age groups studied and methods used to define breakfast. However, the percentage of children who skipped breakfast is similar to national prevalence estimates from the 2011-12 Australian National Nutrition and Physical Activity Survey, where skipping breakfast was defined using two 24-h recalls [25]. In that study 8% of boys aged 4-8 years and 14% of boys aged 9-11 years skipped breakfast on at least one of the 2 days. Among girls 9% of 4-8 year olds and 14% of 9-11 year olds skipped breakfast on at least 1 day [25]. There is no consensus on the best method to assess breakfast. Short questions referring to breakfast consumption on the current day (as used here) are less prone to recall bias but may not capture usual eating patterns. Other studies have used a meal identified as breakfast in a 24-h recall [9]; eating or drinking something at home before school [10]; and eating within 2 hours of getting up [23].

Most studies only assess whether breakfast was eaten and rarely examine the quality of the breakfast. Further research is needed to determine whether certain breakfast types are more beneficial for cognitive performance. A recent systematic review, of studies conducted with children, adolescents and adults, reported there is emerging evidence that breakfast with a low postprandial glycemic response (produces a relatively small increase in blood glucose) has a beneficial effect on cognitive performance [26] but more well-designed studies are needed to determine the ideal breakfast composition. Further research is also needed to determine whether the association between skipping breakfast and academic performance differs by age and whether there are particular ages at which skipping breakfast has greater impacts on academic performance.

## Conclusion

In this national sample of Australian children, few were skipping breakfast at 8-9 years of age. Breakfast skippers had poorer teacher-reported academic performance 2 years later than those who ate breakfast, but the differences in standardized national tests were small and attenuated after adjusting for SES. It is important to use objective measures of academic performance in further studies, otherwise false conclusions may drive unnecessary and costly interventions. The results from our study and well-designed randomized trials examining the effects of school breakfast programs on academic performance [4–6] suggest that, at a population level, public policy to provide breakfast to all elementary-school aged children may not result in improved education outcomes. However, our findings may not apply to populations with a high proportion of children who regularly go without breakfast.

## Additional file

Additional file 1: Figure S1. Time line showing the data collection periods for the Longitudinal Study of Australian children. **Table S1**. Differences between children who were included and excluded from the analyses. **Table S2**. Cross-sectional associations between skipping breakfast aged 8-9 years and teacher-reported academic performance aged 8-9 years (*N* = 2056). **Table S3**. Cross-sectional associations between skipping breakfast aged 8-9 years and teacher-reported behavior aged 8-9 years (*N* = 2054). (PDF 526 kb)

## Abbreviations

LSAC: Longitudinal Study of Australian Children
NAPLAN: National Assessment Program: Literacy And Numeracy
SDQ: Strengths and Difficulties Questionnaire
SES: Socioeconomic status
