# Supplementary material for: Skipping breakfast among 8-9 year old children is associated with teacher-reported but not objectively measured academic performance two years later
Source: BMC Nutr. 2017 Dec 6;3:86. doi: 10.1186/s40795-017-0205-8 (PMC7050735; doi:10.1186/s40795-017-0205-8)
Supplement: Supplementary file 1 — Time line showing the data collection periods for the Longitudinal Study of Australian children. Table S1. Differences between children who were included and excluded from the analyses. Table S2. Cross-sectional associations between skipping breakfast aged 8-9 years and teacher-reported academic performance aged 8-9 years (N = 2056). Table S3. Cross-sectional associations between skipping breakfast aged 8-9 years and teacher-reported behavior aged 8-9 years (N = 2054). (PDF 526 kb) [file 40795_2017_205_MOESM1_ESM.pdf]

Skipping breakfast among 8-9 year old children is associated with teacher-reported but not objectively measured academic performance two years later

Figure S1. Time line showing the data collection periods for the Longitudinal Study of Australian children

Table S1. Differences between children who were included and excluded from the analyses (N=4,331)

Table S2. Cross-sectional associations between skipping breakfast aged 8-9 years and teacher-reported academic performance aged 8-9 years (N=2056)

Table S3. Cross-sectional associations between skipping breakfast aged 8-9 years and teacher-reported behavior aged 8-9 years (N=2054)

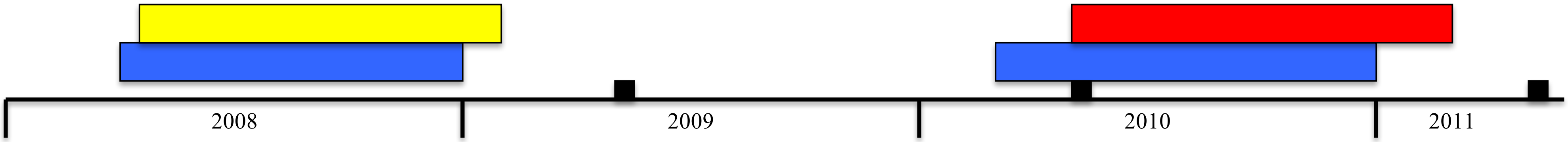

■ Face-to-face interviews with parent/caregiver. Wave 3 April – December 2008 (breakfast measurement 1), Wave 4 March – December 2010.

Blue: Time-use diaries, Wave 3, May 2008 – January 2009 (breakfast measurements 2 and 3).

Yellow: Teacher reports for academic performance and classroom behavior.

Red: NAPLAN: National Assessment Program – Literacy And Numeracy tests, the national standardized school assessment program in Australia, May each year.

Figure S1. Time line showing the data collection periods for the Longitudinal Study of Australian children

Table S1. Differences between children who were included and excluded from the analyses (N=4,331)

Characteristic	Included (n=2,280) *n* (%) or mean ± SD	Excluded (n=2,052) *n* (%) or mean ± SD
Child's sex		
Boy	1181 (51.8)	1032 (50.3)
Girl	1099 (48.2)	1020 (49.7)
Breakfast at interview (N=4,331)[1]		
Ate breakfast	2216 (97.2)	1903 (92.8)
Skipped	64 (2.8)	137 (6.7)
Don't know	0 (0)	11 (0.5)
**Aged 8-9 years**		
Socio-economic status		
Most disadvantaged SES quartile	455 (20.0)	667 (32.6)
Least disadvantaged SES quartile	460 (20.2)	223 (10.9)
Teacher-reported:		
Reading progress (N=3,578)		
Far above/above average	825 (42.4)	516 (31.6)
Average	803 (41.2)	675 (41.4)
Far below/below average	319 (16.4)	440 (27.0)
Mathematics progress (N=3,560)		
Far above/above average	785 (40.6)	482 (29.7)
Average	884 (45.7)	788 (48.5)
Far below/below average	266 (13.8)	355 (21.9)
Overall achievement (N=3,547)		
Far above/above average	786 (40.7)	474 (29.3)
Average	911 (47.2)	790 (48.9)
Far below/below average	233 (12.1)	353 (21.8)
Behavior[2]		
Internalizing problems (N=3,582)	2.34 ± 2.83	2.89 ± 3.29
Externalizing problems (N=3,585)	3.15 ± 3.62	4.15 ± 4.10
Prosocial behavior (N=3,583)	7.75 ± 2.21	7.47 ± 2.33
Year 3 NAPLAN[3] results		
Reading (N=2,930)	437.6 ± 83.2	410.4 ± 83.5
Writing (N=2,931)	438.2 ± 73.4	419.9 ± 73.6
Spelling (N=2,933)	426.6 ± 75.0	409.0 ± 78.2
Grammar (N=2,930)	444.5 ± 86.0	416.4 ± 87.3
Numeracy (N=2,929)	430.3 ± 70.6	408.4 ± 70.4
**Aged 10-11 years**		
Teacher-reported		
Reading progress (N=3,240)		
Far above/above average	837 (43.7)	437 (33.0)
Average	785 (41.0)	589 (44.5)
Far below/below average	293 (15.3)	299 (22.6)
Mathematics progress (N=3,209)		
Far above/above average	784 (41.3)	390 (29.8)

Average	836 (44.0)	614 (46.9)
Far below/below average	279 (14.7)	306 (23.4)
Overall achievement (N=3,227)		
Far above/above average	812 (42.6)	410 (31.1)
Average	870 (45.6)	653 (49.5)
Far below/below average	226 (11.8)	256 (19.4)
Behavior[2]		
Internalizing problems (N=3,268)	2.29 ± 2.97	2.51 ± 3.02
Externalizing problems (N=3,267)	2.88 ± 3.54	3.55 ± 3.87
Prosocial behavior (N=3,268)	7.93 ± 2.20	7.72 ± 2.18
Year 5 NAPLAN[3] results		
Reading (N=3,841)	518.7 ± 79.7	492.0 ± 82.9
Writing (N=3,828)	501.8 ± 68.6	485.9 ± 71.0
Spelling (N=3,835)	501.8 ± 66.2	485.3 ± 72.9
Grammar (N=3,835)	527.7 ± 80.9	501.6 ± 88.3
Numeracy (N=3,820)	512.1 ± 72.1	489.7 ± 73.3

[2]NAPLAN, National Assessment Program – Literacy And Numeracy.

[1] Breakfast consumption reported by the parent/caregiver at the face-to-face interview was assessed using the question "Did <study child> eat breakfast today?". This measure of breakfast consumption was used for this analysis as nearly all participants (those included and excluded from the main analysis) had data for this question.

[2] Values are the unadjusted mean ± SD score for the three scales of the Strengths and Difficulties Questionnaire. Better behavior is indicated by lower scores for internalizing problems (range 0 – 18) and externalizing problems (range 0 – 20) and higher scores for prosocial behavior (range 0 – 10).

[3] Values are the unadjusted mean ± SD for each domain of the NAPLAN assessments. Possible score range 0-1,000, higher scores indicate better academic performance.

Table S2. Cross-sectional associations between skipping breakfast aged 8-9 years and teacher-reported academic performance aged 8-9 years (N=1,953)

Outcome and category of skipping breakfast	Above average[1] *n* (%)	Average[1] *n* (%)	Below average[1] *n* (%)	Model 1[2] PR (95% CI)	Model 2[3] PR (95% CI)	Model 3[4] PR (95% CI)
Reading progress						
Never skipped	742 (42.7)	716 (41.2)	218 (16.2)	1.00 (ref)	1.00 (ref)	1.00 (ref)
≥1 skips	83 (39.9)	87 (41.8)	38 (18.3)	1.08 (0.98, 1.20)	1.08 (0.98, 1.19)	1.05 (0.96, 1.15)
P-value				0.10	0.10	0.25
Mathematics progress						
Never skipped	699 (40.6)	783 (45.4)	242 (14.0)	1.00 (ref)	1.00 (ref)	1.00 (ref)
≥1 skips	84 (40.6)	99 (47.8)	24 (12.0)	1.03 (0.93, 1.14)	1.04 (0.94, 1.15)	1.04 (0.94, 1.15)
P-value				0.54	0.44	0.41
Overall achievement						
Never skipped	696 (40.6)	810 (47.3)	208 (12.1)	1.00 (ref)	1.00 (ref)	1.00 (ref)
≥1 skips	83 (40.9)	97 (47.8)	23 (11.3)	1.05 (0.95, 1.17)	1.05 (0.95, 1.17)	1.04 (0.94, 1.14)
P-value				0.33	0.32	0.46

[1] Comparisons are to other children of the same grade level. Below average = below/far below average; Above average = above/far above average.
[2] Model 1: adjusted for sex and age at time of the parent interview.
[3] Model 2: adjusted for sex, age at time of the parent interview and SES (measured at Wave 3).
[4] Model 3: Model 2 plus the following additional covariates *reading progress* – smoking status of primary caregiver; *mathematics progress* – financial hardship, two-parent home, teacher reported pro-social behavior; *overall achievement* – financial hardship, two-parent home, smoking status of primary caregiver, self-reported health of primary caregiver, teacher reported prosocial behavior.

Table S3. Cross-sectional associations between skipping breakfast aged 8-9 years and teacher-reported behavior aged 8-9 years (N=1952)

Behavior subscale and category of skipping breakfast	*n*	Mean ± SD[1]	Model 1[2] Diff (95% CI)[5]	Model 2[3] Diff (95% CI)[5]	Model 3[4] Diff (95% CI)[5]
Internalizing problems					
Never skipped	1726	2.47 ± 3.28	0 (ref)	0 (ref)	0 (ref)
≥1 skips	206	2.86 ± 4.41	0.10 (-0.47, 0.67)	0.04 (-0.50, 0.57)	0.13 (-0.65, 0.38)
P-value			0.73	0.90	0.61
Externalizing problems					
Never skipped	1726	3.47 ± 4.42	0 (ref)	0 (ref)	0 (ref)
≥1 skips	206	3.75 ± 5.00	0.24 (-1.75, 2.23)	-0.16 (-1.92, 1.60)	-0.89 (-2.48, 0.69)
P-value			0.81	0.86	0.24
Prosocial behavior					
Never skipped	1743	7.66 ± 2.44	0 (ref)	0 (ref)	----[6]
≥1 skips	209	7.37 ± 2.47	-0.21 (-0.46, 0.04)	-0.17 (-0.41, 0.07)	----[6]
P-value			0.10	0.16	

[1] Values are the unadjusted mean ± SD score for the three scales of the Strengths and Difficulties Questionnaire. Better behavior is indicated by lower scores for internalizing problems (range 0 – 18) and externalizing problems (range 0 – 20) and higher scores for prosocial behavior (range 0 – 10).
[2] Model 1: adjusted for sex and age at interview.
[3] Model 2: adjusted for sex, age at interview and SES (measured at Wave 3)
[4] Model 3: Model 2 plus additional adjustments for *internalizing problems* – two-parent home, financial hardship, self-reported health of primary caregiver, reading progress; *externalizing problems* – two-parent home, financial hardship, self-reported health of primary caregiver, reading progress.
[5] Differences between breakfast skippers and breakfast eaters were calculated using linear regression.
[6] There was no model 3 for prosocial behavior as none of the additional covariates changed the coefficient of the covariate for skipping breakfast by at least 10%.
